# Severe hypoxia induces chemo-resistance in clinical cervical tumors through MVP over-expression

**DOI:** 10.1186/1748-717X-4-29

**Published:** 2009-08-06

**Authors:** Pedro C Lara, Marta Lloret, Bernardino Clavo, Rosa M Apolinario, Luis Alberto Henríquez-Hernández, Elisa Bordón, Fausto Fontes, Agustín Rey

**Affiliations:** 1 Radiation Oncology Department, Hospital Universitario de Gran Canaria Dr. Negrín, Spain; 2 Canary Institute for Cancer Research (ICIC), Spain; 3 Clinic Sciences Department of Las Palmas de Gran Canaria University (ULPGC), Spain; 4 Pathology Department, Hospital Universitario de Gran Canaria Dr. Negrín, Spain

## Abstract

Oxygen molecule modulates tumour response to radiotherapy. Higher radiation doses are required under hypoxic conditions to induce cell death. Hypoxia may inhibit the non-homologous end-joining DNA repair through down regulating Ku70/80 expression. Hypoxia induces drug resistance in clinical tumours, although the mechanism is not clearly elucidated. Vaults are ribonucleoprotein particles with a hollow barrel-like structure composed of three proteins: major vault protein (MVP), vault poly(ADP-ribose) polymerase, and telomerase associated protein-1 and small untranslated RNA. Over-expression of MVP has been associated with chemotherapy resistance. Also, it has been related to poor outcome in patients treated with radiotherapy alone. The aim of the present study was to assess the relation of Major Vault Protein expression and tumor hypoxia in clinical cervical tumors. MVP, p53 and angiogenesis, together with tumor oxygenation, were determined in forty-three consecutive patients suffering from localized cervix carcinoma. High MVP expression was related to severe hypoxia compared to low MVP expressing tumors (p = 0.022). Tumors over-expressing MVP also showed increased angiogenesis (p = 0.003). Besides it, in this study we show for the first time that severe tumor hypoxia is associated with high MVP expression in clinical cervical tumors. Up-regulation of MVP by hypoxia is of critical relevance as chemotherapy is currently a standard treatment for those patients. From our results it could be suggested that hypoxia not only induces increased genetic instability, oncogenic properties and metastatization, but through the correlation observed with MVP expression, another pathway of chemo and radiation resistance could be developed.

## Introduction

Growing cancers often acquire an increasing number of genetic alterations. Such genetic changes, including chromosomal translocation, gene amplification, intragenic mutation, and gene silencing, are responsible for the activation of oncogenes and the inactivation of tumour-suppressor genes [1]. How cancer cells acquire genetic instability remains unclear. Exposure of cells to adverse conditions like hypoxia can lead to genome alterations, enhancing the progression potential of tumor cells and resistance to oncological treatments [1]. Hypoxia may lead to conditions that causes increased spontaneous damage to DNA or inhibit DNA repair processes, impair DNA repair and cause tumor progression by altered p53 expression and increased angiogenesis [2,3]. Deregulation of DNA repair pathways can contribute to the phenomenon of hypoxia-induced genetic instability within the tumor [4]. Hypoxia is measured in clinical tumors by several techniques, including the Eppendorf polarographic method [2,5]. In cervical cancer patients, hypoxia is commonly associated to a lesser response to treatment and lower survival rates [6,7]. Hypoxic tumors have a significant higher probability of relapse and death [7] and they are resistant to chemotherapy [8]. Chemo-resistance would be mediated by up-regulation of Major Vault Protein (MVP) through the Hypoxia-inducible factor 1 (HIF-1) as shown in previously studies performed in vitro [9]. Hypoxia inhibits the non-homologous end joining (NHEJ) DNA repair through down-regulating Ku70/80 expression, combined with increased angiogenesis and altered p53 expression [2]. Cervical tumors over-expressing MVP also showed down-regulation of Ku70/80 and BAX [10]. MVP over-expression has been associated with a suppression of NHEJ repair, and subsequent genomic instability [10]. These mechanisms would be responsible for tumor progression in cervical carcinoma. Moreover, MVP over-expression was associated to reduced long-term local control in patients who achieved clinical complete response to radio-chemotherapy [11]. The aim of the present study was to assess the relation between the expression of the Major Vault Protein and tumor hypoxia in clinical cervical tumors.

## Methods

Forty-three consecutive patients suffering from localized cervix carcinoma were prospectively included in this study from July 1997 to September 2001 [2]. Patients were diagnosed and treated by definitive radiation at the Hospital Universitario Materno-Infantil, at the Hospital Universitario Dr. Negrín and at the Hospital Universitario Insular in Las Palmas de Gran Canaria (Spain). Written informed consent was given previously. The study was approved by the Research and Ethics Committee of our institution. The mean age of the patients was 49.48 ± 12.79 years (median 48, range 29–81 years). Fourteen patients had stage I disease, 22 stage II and 7 stage III-IVA. MVP expression was studied by immunohistochemistry in paraffin-embedded 4 μm sections incubated for the specific primary antibody (MVP, Neomarkers CA, USA). A secondary biotinated antibody (Dako Detection Kit, LSBA) was incubated for 30 minutes, and peroxidase-streptavidin-biotin complex (Dako) was used afterward. Staining was revealed by using diaminobenzidine tetra-hydrochloride substrate (DAB Chromogen; Dako), followed by light counterstaining with Harris hematoxylin as previously described [10]. Data of p53 and angiogenesis, estimated by CD-31 staining, were obtained from our files [2]. Paraffin-embedded tissues from tumor biopsies were available from all patients, and the most representative tumor block was used for immunohistochemical analysis. Blocks were handled as previously described and then incubated for the specific secondary antibody (p53, Clon:DO-7, Novocastra Laboratories Ltd., Newcastle upon Tyne, UK; CD-31 Clon:JC/70A, Dako, Carpintería, CA, USA) [2]. The primary antibody was omitted in one section as a negative control in each set of slides. As a positive control, a strong positive tumor for the oncoprotein was used. Tumor oxygenation was measured by an Eppendorf device following standard criteria as previously described [2,12] using a polarographic probe system "pO2 Histograph" (Eppendorf AG, Hamburg, Germany). For each set of measurements obtained from tumor, 200 single pO2 values were recorded using at least 6 different electrode tracks. Tumor hypoxia data were reanalyzed for detecting cases of severe hypoxia and the percentage of pO2 values < 2.5 mmHg were obtained from the pooled data and for each individual. Assessment of immunostaining or tumor oxygenation result was blinded to knowledge of the clinical outcome of the patient. Statistical analysis was performed by SPSS 15.0 software.

## Results

**Figure 1:**
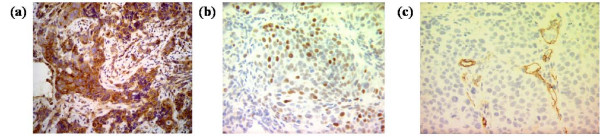
**Representative immunostaining of MVP (a), p53 (b) and micro-vessels (c)**.

**Figure 2:**
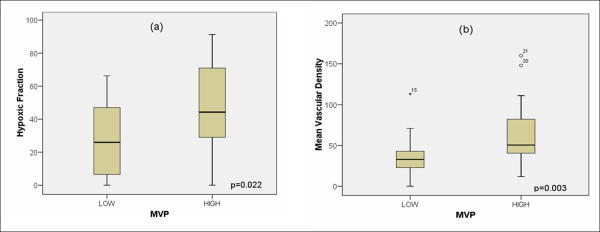
**Relationship between (a) MVP and hypoxic fraction (HF 2.5) and (b) mean vascular density**.

All immunohistochemical markers and hypoxia values were known in all 43 cases (Figure 1). MVP expression was considered low (negative/slightly positive) in 23 cases and high (strongly positive) in 20 cases. Data of mean vascular density (MVD) and p53 expression were obtained from our files [2] (Table 1). MVD was 49.62 ± 33.98% (median 41%, range 0–160). P53 expression showed a mean value of 39.15 ± 27.62% (median 35%, range 0–92%). Tumor hypoxia was also known in all patients. Mean tumor hypoxic fraction <2.5 mmHg (HF 2.5) values were 35.89 ± 26.80 (median 35.20%, range 0–91.30%). MVP expression was independent of clinical and histological variables, except for adenocarcinoma tumors. In fact adenocarcinoma tumors (5 cases) included in the present study over-expressed MVP versus 15 out of 38 squamous cancers (p = 0.011). Besides, high MVP expression was related to severe hypoxia as determined by higher hypoxic fractions HF (2.5) (45.82 ± 28.00%) compared to low MVP expressing tumors (27.26 ± 22.96%) (p = 0.022) (Figure 2a). Tumors over-expressing MVP also showed increased angiogenesis (65.41 ± 38.38) compared to low expressing cases (35.89 ± 22.55) (p = 0.003) (Figure 2b). MVP expression was independent of p53 protein expression.

**Table 1:** Characteristics of the patients in the study

Characteristics	All patients (n = 43)	MVP low (n = 23)	MVP high (n = 20)	P value
Age	49.48 ± 12.79	49.47 ± 13.68	49.50 ± 12.04	
	(29–81)	(29–81)	(32–72)	0.325
Stage				
I	14	5	9	
II	22	13	9	
III	7	5	2	0.228
Histology				
Epidermoid	38	23	15	
Adenocarcinoma	5	0	5	0.011
Grade				
I	5	3	2	
II	19	10	9	
III	19	10	9	0.952
p53	39.15 ± 27.62	37.53 ± 28.04	41.02 ± 27.74	
	(0–92)	(0–92)	(0–81)	0.685
Vascular density	49.62 ± 33.98	35.89 ± 22.55	65.41 ± 38.38	
	(0–160)	(0–113)	(12–160)	0.003
Hypoxic fraction	35.89 ± 26.80	27.26 ± 22.96	45.82 ± 28.00	
	(0–91.30)	(0–66.30)	(0–91.30)	0.022
Median pO_2_	7.61 ± 8.98	7.84 ± 7.85	7.36 ± 10.34	
	(0–41.90)	(0–24.30)	(0–41.90)	0.863

Mean ± standard deviation and range are included as well as p53, vascular density, hypoxic fraction and median of pO_2_

## Discussion

In this study we show for the first time that severe tumor hypoxia is related to high MVP expression in clinical cervical tumors. MVP is ubiquitously expressed and, besides chemotherapy resistance, it has been implicated in the regulation of several cellular processes including transport mechanisms, signal transmissions and immune responses [13]. Previous studies have demonstrated that vaults are up-regulated in different multidrug resistant cancer cell lines [14] and resistance models [15,16]. Increased levels of MVP have been reported in numerous cell lines after selection with a wide panel of cytostatic drugs (e.g. doxorubicin, methotrexate, vincristine or cisplatin) [17]. By contrast, tumour necrosis factor-either applied externally or after gene transduction, led to down-regulation of MVP transcription [18]. There are several publications concerning to the relationship between MVP expression and drug resistance in clinical oncology [19-22]. The role of MVP in clinical outcome after radio-chemotherapy in cervical carcinoma [11] and other cancers [23] has been reported. MVP seems to down-regulate the pro-apoptotic gene BAX through its relation with Ku70/80. Ku70/80 are key genes in the NHEJ repair pathway for radiation-induced DNA double strand breaks. Expression of Ku70/80 has been related to survival in patients treated with x-rays [24,25]. Ku70/80 is a central regulator of apoptosis by interacting with BAX [26] and BCL-2, which in turn has been shown to suppress Ku, thus inhibiting NHEJ repair [27]. In the clinical setting, up-regulation of MVP by hypoxia is of critical relevance because chemotherapy is currently a standard treatment for those patients. In the other hand, hypoxia inhibits the NHEJ DNA repair through down-regulating Ku70/80 expression [2]. Preclinical studies about the role of hypoxia in cancer cells showed that reduction of pO_2 _is a favoring factor to increase chemo-resistance [8,28]. In cancer, hypoxia is an adverse prognostic indicator associated with tumor progression and resistance to therapy [29]. Cellular drug delivery and uptake in hypoxic areas are affected by hypoxia. Some chemotherapeutic drugs require oxygen to generate free radicals that contribute to cytotoxicity. Hypoxia induces cellular adaptations that compromise the effectiveness of chemotherapy. Moreover, the expression of several genes controlling tumor cell survival is regulated by hypoxia (e.g., growth factors governing the formation of new blood vessels and hypoxia-responsive transcription factors modulating the expression of genes). The transcription factor Hypoxia-inducible factor 1 (HIF-1) is one of the principal mediators of homeostasis in human tissues exposed to hypoxia. It is implicated in virtually every process of rapid gene expression in response to low oxygen levels [30]. HIF-1alpha is over-expressed in the majority of common human cancers and their metastases, due to the presence of intratumoral hypoxia and as a result of mutations in genes encoding oncoproteins and tumor suppressor genes [31,32]. Whether in clinical tumors this chemo-resistance can be reverted by HIF-1 inhibitors deserves to be studied [9]. Pharmacologic manipulation of HIF-1 levels may provide a novel therapeutic approach to diseases like cancer, especially in combination with anti-angiogenic agents [33] that would further reduce tumour oxygenation. Our previously clinical results showed a close relation of clinical hypoxia to increased angiogenesis and in a lesser extent to p53 oncoprotein alteration [2]. Clinical outcome in patients suffering different types of tumours mainly treated by radiation (i.e., cervical and head & neck cancers) depends, at least in part, of those parameters. An increased genetic instability, oncogenic properties, resistance to treatment and increased ability to metastization are expected.

From our results it could be suggested that hypoxia not only induces increased genetic instability, oncogenic properties and metastatization, but through the correlation observed with MVP expression, another pathway of chemo-resistance could be developed.

## Abbreviations

HIF-1: Hypoxia-inducible factor 1; MVD: Mean vascular density; MVP: Major Vault Protein; NHEJ: non-homologous end joining.

## Conflict of interests

The authors declare that they have no competing interests.

## Authors' contributions

PCL has been involved in conception and design of the study as well as in drafting the manuscript, and has given final approval of the version to be published. MLl has made the measurements of tumour hypoxia and has treated all patients. BC has made the measurements of tumour hypoxia. RMA has made the angiogenesis studies. LAHH has been involved in the writing of the manuscript and type of packaging likewise in the submission process. EB has made the MVP studies. FF has made the p53 studies. AR has reviewed and overlooked all the immunohistochemistry experiments.
